# Rotavirus vaccine clinical trials: a cross-sectional analysis of clinical trials registries

**DOI:** 10.1186/s13063-022-06878-6

**Published:** 2022-11-17

**Authors:** Duduzile Ndwandwe, Sinazo Runeyi, Lindi Mathebula, Charles Wiysonge

**Affiliations:** 1 Cochrane South Africa, South African Medical Research Council, Parow Valley, 7501 Cape Town, South Africa; 2 HIV and Other Infectious Diseases Research Unit, South African Medical Research Council, Durban, 4091 South Africa; 3 Division of Epidemiology and Biostatistics, Faculty of Medicine and Health Sciences, Stellenbosch University, Tygerberg, 7505 Cape Town, South Africa; 4 School of Public Health and Family Medicine, Faculty of Health Sciences, University of Cape Town, Observatory, 7925 Cape Town, South Africa

**Keywords:** Rotavirus vaccine, Clinical trial registry, Cross-sectionally analysis

## Abstract

**Background:**

Rotavirus is a primary infectious virus causing childhood diarrhoea and is associated with significant mortality in children. Three African countries (Nigeria, the Democratic Republic of Congo, and Angola) are among the five countries that account for 50% of all diarrheal-related deaths worldwide. This indicates that much needs to be done to reduce this burden. The World Health Organization International Clinical Trial Registry Platform (WHO ICTRP) is a global repository for primary registries reporting on clinical trials. This study aimed to identify and describe planned, ongoing, and completed rotavirus vaccine trials conducted globally.

**Methods:**

We searched WHO-ICTRP on 17 June 2021 and conducted a cross-sectional analysis of rotavirus studies listed in the database. Data extraction included trial location, participant age, source of the trial record, trial phase, sponsor, and availability of results. We used the Microsoft Excel 365 package to generate descriptive summary statistics.

**Results:**

We identified 242 rotavirus vaccine trials registered from 2004 to 2020. Most of these trials were registered retrospectively, with only 26% of the rotavirus vaccine trials reporting the availability of results in their registries. Most of the trials are studying children aged less than 5 years. The recruitment status for these trials is currently shown in the WHO-ICTRP as “not recruiting” for 80.17% of trials, “recruiting” for 11.57% of trials recruiting, and unknown for 6.61% of trials. The continents in which these rotavirus vaccine trials have recruitment sites in Asia (41%) and North America (20%), with the maximum number of trials in the clinical trial registries coming from India (21%) and the USA (11%) with most being sponsored by the pharmaceutical industry. Our analysis shows that only 26% of the rotavirus vaccine trials report the availability of results in their registries.

**Conclusions:**

Mapping rotavirus vaccine clinical trial activity using data from the WHO ICTRP beneficial provides valuable information on planned, ongoing, or completed trials for researchers, funders, and healthcare decision-makers. Despite the high rotavirus disease burden in low- and middle-income countries, including Africa, there is minimal clinical trial activity related to the condition on the continent. The clinical trial registries as a valuable tool to share interim results of the trials.

## Introduction

Globally, rotavirus remains the most common cause of diarrheal disease in infants and children under the age of 5 years, particularly in low-income countries, and continues to significantly impact childhood morbidity and mortality [1]. Before the introduction of vaccines, rotavirus infection accounted for about 37% of hospitalisations annually and over 200,000 deaths each year, with four countries (Nigeria, India, Pakistan, and the Democratic Republic of Congo) accounting for approximately half (49%) of all estimated global diarrheal related deaths in 2013 [2].

The introduction of the rotavirus vaccine in national immunisation programmes has averted the incidence of gastroenteritis [3]. Besides this outstanding achievement, the Central African Republic, South Sudan, Gabon, Chad, Guinea, Equatorial Guinea, and Comoros have yet to introduce the rotavirus vaccine, which translates to a 10–30% reduction in diarrhoeal incidence rate. In comparison, Nigeria and Somalia have noted an approximate increase of 10% in rotavirus vaccine uptake [4]. Nine countries in sub-Saharan Africa are yet to introduce the rotavirus vaccine. These countries include the Central African Republic, Chad, Comoros, Equatorial Guinea, Gabon, Guinea, Nigeria, Somalia, and South Sudan; Chad, the Central African Republic, Comoros, Guinea, Somalia, and South Sudan are all Gavi-eligible countries [4]. This led to a hundred countries introducing rotavirus vaccines in their national immunisation programmes in 2019. Additionally, six low-income countries are being approved for funding support for vaccine introduction from Gavi, the Vaccine Alliance. They are awaiting a national introduction, with another 13 countries preparing to introduce themselves independently of Gavi support [2].

Rotaviruses are non-enveloped viruses belonging to the Reoviridae family, characterised by the double-stranded RNA (dsRNA) segments which can be classified into ten distinct groups (A–J). Rotavirus A is the most common species, accounting for more than 90% of human rotavirus infections [1]. The rotavirus genome encodes six structural genes (*VP1*, *VP2*, *VP3*, *VP4*, *VP6*, *VP7*) and six non-structural genes (NPD1–NSP5), which determine host specificity, cell entry, and enzymatic functions in their mature state [1]. Rotavirus is shed in stools and mainly transmitted by faecal-oral contact, contaminated surfaces, and respiratory droplets [5], thus infecting the small intestine and causing watery diarrhoea.

Besides young children being affected by rotavirus, a few outbreaks among adults have been reported in the literature [6]. An example is rotavirus infection in immunocompromised adults, varying from symptomless to severe [6]. The World Health Organization (WHO) recommended the rotavirus vaccines for priority inclusion in routine immunisation programmes as part of a preventative package for all infants in response to the urgent need for interventions to reduce preventable deaths caused by rotavirus infections [1].

Many countries have seen significant reductions in disease burden following the introduction of rotavirus vaccines. These vaccines had undergone clinical trials and were safe and effective in preventing severe acute gastroenteritis in children [7]. In 2006, two live oral vaccines, Rotarix® (GlaxoSmithKline Biologicals SA, Rixensart, Belgium) and RotaTeq® (Merck & Co., Inc., West Point, PA, USA), were licenced for use in infants in several countries. Rotarix® is a 2-dose monovalent human rotavirus vaccine, and RotaTeq® is a 3-dose pentavalent bovine-human reassortant rotavirus vaccine [2]. These two vaccines have been incorporated into national immunisation programmes in over 80 countries worldwide [2]. While rotavirus vaccines have been highly effective in preventing diseases associated with rotavirus infection in high-income countries, their efficacy and effectiveness are substantially lower in low- and middle-income countries where the incidence of the disease is the highest [7].

The occurrence of the disease is further exacerbated by living conditions and environmental factors such as access to safe drinking water, sanitation, higher hygiene standards, poverty, nutrition, and timely access to healthcare [7]. This can diminish the efficiency of vaccines and hence poorer health outcomes for the unprivileged, especially those in developing countries. More efficient vaccines are thus needed to have the full benefit of receiving vaccines, especially where they are needed the most.

New live oral and non-replicating vaccine candidates continue to be developed to improve vaccine efficacy in developing countries [8]. As of 2018, several new rotavirus vaccines have obtained national licensure in addition to the globally available Rotarix® and RotaTeq® WHO-prequalified vaccines [9]. Rotavac (Bharat Biotech, Hyderabad, India) and ROTASIL (Serum Institute of India PVT. LTD., Pune, India) was prequalified by WHO and are currently only in use in India. Rosavinavin-M1 (PolyVac) is manufactured and licenced in Vietnam [9]. This expanding product landscape will ensure adequate global supply and vaccine diversity and will thus provide opportunities to optimise immunisation programmes [10].

We provide a cross-sectional survey analysis of the rotavirus vaccine clinical trials listed in the International Clinical Trials Registry Platform.

## Methods

### Study design

The World Health Organization International Clinical Trials Registry Platform (ICTRP) is a global initiative that aims to make information about all clinical trials involving human beings publicly available. We report a cross-sectional survey analysis of the rotavirus vaccine clinical trials listed in the International Clinical Trials Registry Platform. The ICTRP was searched using the term “rotavirus vaccine” on 17 June 2021. The data was downloaded and converted to an excel file for analysis. The downloaded data included the 24 data item fields that the ICTRP collects from primary registers and data providers. The collective data search had all records available in the ICTRP platform with no date limitation. A descriptive analysis was conducted on the search outputs.

### Data management and analysis

#### Data extraction

Data were extracted from the ICTRP and exported into an Excel spreadsheet by one researcher (DN). All records were quality-checked by a second researcher (SR) to ensure that rotavirus vaccine clinical trials were included. The rotavirus vaccine trials considered for the analysis were randomised controlled trials in humans evaluating the safety, immunogenicity, efficacy, and effectiveness. In each record of the included trials, the following data items were used for analysis: date of registration, anticipated last follow-up date, actual last follow-up date, retrospective and prospective registration, disease researched, location of the trial, location of principal investigator, intervention type, age range of participants, and funding source. We used the Microsoft Excel 365 package to generate descriptive summary statistics.

## Results

**Fig. 1:**
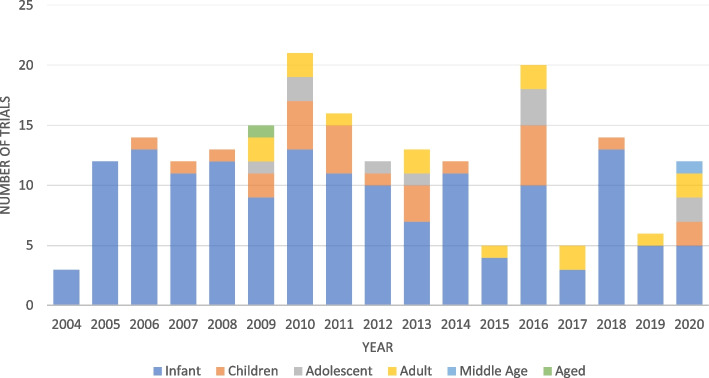
Distribution of rotavirus vaccine trial registration by age from 2004 to 2020

A total of 242 records were identified in the ICTRP for clinical trials reporting on the rotavirus vaccine. These trials were registered in clinical trial registries from 2004, with the last registration when data was downloaded being the year 2020. We assessed the distribution of the age of the participants over the years of the rotavirus trials registration after we had defined the age categories as follows: infants (1–23 months), children (2–12 years), adolescents (13–18 years), adult (19–105 years), middle-aged (45–64 years), and aged (above 65 years). Figure 1 shows that most of the rotavirus vaccine trials have been conducted in infants throughout the trial registration. Most of these trials were registered retrospectively, with only 26% of the rotavirus vaccine trials reporting the availability of results in their registries.

ICTRP is a one-stop platform in which WHO primary registries records and ClinicalTrials.gov can be accessed. We found the following registries which listed rotavirus vaccine trials: Australian New Zealand Clinical Trials Registry (ANZCTR), Chinese Clinical Trial Registry (ChiCTR), Clinical Trials Registry - India (CTRI), EU Clinical Trials Register (EU-CTR), Japan Primary Registries Network (JPRN), Netherlands National Trial Register (NTR), Pan African Clinical Trial Registry (PACTR), and Peruvian Clinical Trial Registry (REPEC). We show that 65% of the clinical trials reporting on rotavirus vaccines are registered with ClinicalTrials.gov (Table 1).

**Table 1:** Number of clinical trials in registries

Registry	Number	Per cent
ANZCTR	5	2.07%
ChiCTR	3	1.24%
ClinicalTrials.gov	155	64.05%
CTRI	33	13.64%
EU-CTR	28	11.57%
ISRCTN	9	3.72%
JPRN	4	1.65%
NTR	1	0.41%
PACTR	2	0.83%
REPEC	2	0.83%
Total	**242**	**100.00%**

The recruitment status of a trial indicates the progress of the trial. We assessed the trial status of the rotavirus vaccine trials listed in the International Clinical Trials Registry Platform. We found that 80% of the rotavirus vaccine trials are not recruiting, with only 11% of the vaccine trials currently recruiting. We also noted that some registries list their status as authorised, which indicates in progress (1%) and other trial records with status listed as not applicable (6%) (Table 2).

**Table 2:** Trials by overall status

Recruitment status	Number	Per cent
Authorised	4	1.65%
Not available	16	6.61%
Not recruiting	194	80.17%
Recruiting	28	11.57%
Total	**242**	**100.00%**

The clinical trial stage of the rotavirus vaccine trials allows an overview of the rotavirus vaccine pipeline. Our data show that 33% of the rotavirus vaccine trials are in the phase 3 clinical trials stage, and 11% are in the phase 4 clinical trial phase. We also found a considerable number of the rotavirus vaccine trials (33%), which either did not indicate the phase of the trials or were listed as “not applicable” (Table 3).

**Table 3:** Clinical trials by phase

Phase	Number	Per cent
Phase 1	23	9.50
Phase 1/phase 2	8	3.31
Phase 2	23	9.50
Phase 2/phase 3	2	0.83
Phase 3	80	33.06
Phase 4	27	11.15
Not applicable	39	16.12
Not indicated	40	16.53
Grand total	**242**	**100.00**

**Fig. 2:**
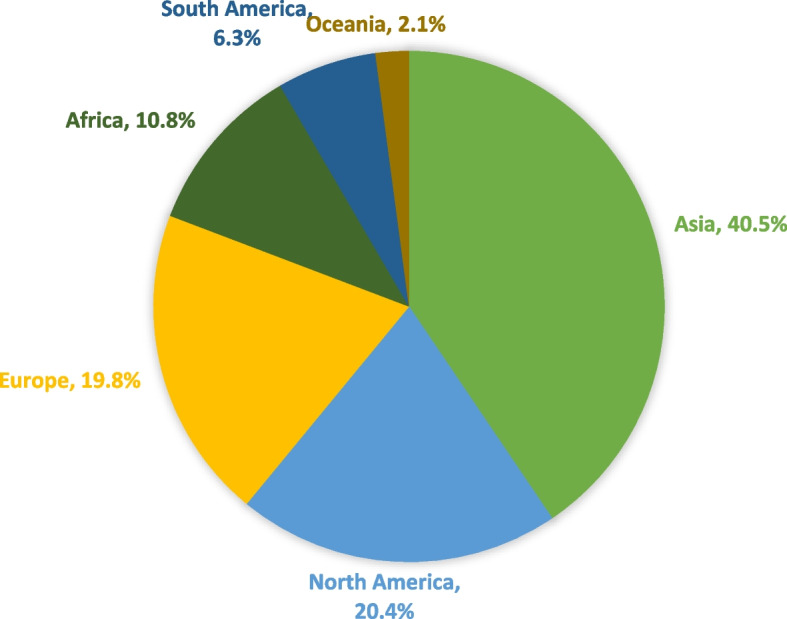
Geographic distribution of rotavirus vaccine clinical trials

In our analysis, we first evaluated the geographic distribution of the rotavirus vaccine trials. We show that most of the rotavirus vaccine trials are conducted in Asia (41%), followed by North America (20%) (Fig. 2).

**Fig. 3:**
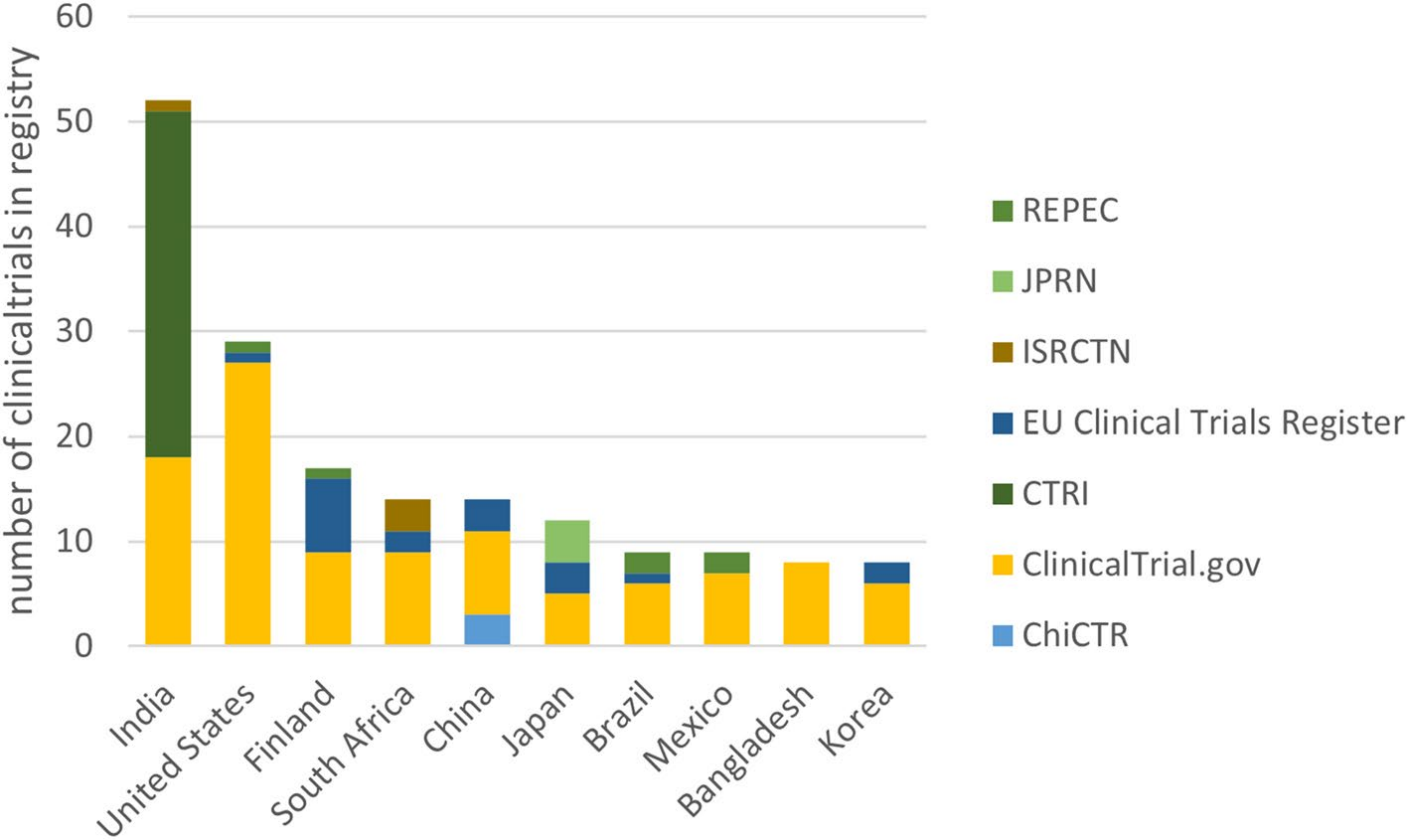
Clinical trial registries and the recruitment site location of rotavirus vaccine trials. Clinical trial registries: Australian New Zealand Clinical Trials Registry (ANZCTR), Chinese Clinical Trial Registry (ChiCTR), Clinical Trials Registry - India (CTRI), EU Clinical Trials Register (EU-CTR), Japan Primary Registries Network (JPRN), Netherlands National Trial Register (NTR), Pan African Clinical Trial Registry (PACTR), Peruvian Clinical Trial Registry (REPEC), ClinicalTrials.gov, and International Standard Randomised Controlled Trial Number (ISRCTN)

We wanted to understand the countries with the most rotavirus vaccine trials conducted. In total, we identified 55 countries across the globe which have rotavirus vaccine trials. Some have multiple sites within the same country, while others have only single sites within these countries. We compared the source of the registry and the country where these trials were conducted. We present the top countries conducting rotavirus vaccine trials and compare the clinical trial registry with the number of vaccine trials in a country. We found that the USA and India had the most trials registered with ClinicalTrials.gov and CTRI (32%) (Fig. 3).

The sponsor of a clinical trial has a responsibility to register a trial. We categorised the sponsors into categories after assessing each record. Therefore, we evaluated the sponsors of the rotavirus vaccine trials and found that the pharmaceutical industry sponsors almost 70% (Table 4).

**Table 4:** Number of trials registered by sponsor type

Type of sponsor	Number	Per cent
Governmental	28	11.6
University	25	10.3
Pharmaceutical company	167	69.0
Self-funding	2	0.8
No funding	1	0.4
Funding agency	1	0.4
Research institute	10	4.1
Non-profit organisation	3	1.2
Hospital	5	2.1
Total	242	100%

## Discussion

We conducted a descriptive analysis of the rotavirus vaccine clinical trials in the International Clinical Trials Registry Platform. In 2013, an estimated 214,664 deaths were attributed to rotavirus infection in developing countries, and in the absence of rotavirus vaccine introduction, 38% of all hospitalised diarrhoea cases were among children. In 2006, the World Health Organization’s (WHO) Strategic Advisory Group of Experts (SAGE) first recommended the inclusion of rotavirus vaccines into national immunisation programmes in Europe and the Americas, and later in 2009, SAGE recommended the integration of rotavirus vaccine into all immunisation programmes worldwide [11].

The risk of intussusception was first identified in the USA in 1999 with the RotaShield vaccine, with increasing cases in children in many other countries necessitated ongoing clinical research on rotavirus vaccines.

Our data show that vaccine clinical research has been ongoing since 2004, with most of the trials in phase 4 clinical trial stages involving infants and children. The benefit of continual post-marketing surveillance after introducing vaccines provides further data on how the vaccines perform in real life. The data from the phase 4 studies from high-income and middle-income countries have detected a temporally limited but significant increase in the risk of intussusception in the 1–7 days following administration of Rotarix or RotaTeq, on the order of 1 to 6 excess cases per 100,000 infants vaccinated. The contextual differences such as access to healthcare and rotavirus vaccine effectiveness between high-income and low-income countries need to be assessed separately as results from one setting cannot be generalised to the other. Also, intussusception’s baseline incidence and epidemiology vary by country and location [9].

We report on the 242 trials registered in clinical trial registries from 2004 to 2020 based on data from the ICTRP. No rotavirus vaccine trials were recorded in the year 2021 owing to the COVID-19 pandemic, where research activities focused on finding vaccines and therapeutics to curb the rapidly spreading SARS-Nov-2 infections. Most of these trials were registered retrospectively, while the WHO ICTRP advocates for prospective trial registration [12–14]. This shows that there is a need for registries to continue to promote prospective trial registration.

Our data show 11% of rotavirus vaccine trials in phase 4, the post-licensure clinical trial stage. Burnett et al. reported a systematic review that evaluated post-licensure vaccine effectiveness data stratified by a country’s childhood mortality rates [15]. The review found that Rotarix vaccine effectiveness against laboratory-confirmed rotavirus among children younger than 12 months old was 86% (95% CI 81–90) in low-mortality countries, 77% (66–85) in medium-mortality countries, and 63% (54–70) in high-mortality countries. Furthermore, Rotarix vaccine effectiveness among children aged 12–23 months was 86% (81–90) in low-mortality countries, 54% (23–73) in medium-mortality countries, and 58% (38–72) in high-mortality countries [15]. RotaTeq vaccine effectiveness among children younger than 12 months was 86% (76–92) in low-mortality countries and 66% (51–76) in high-mortality countries. RotaTeq vaccine effectiveness among children aged 12–23 months was 84% (79–89) in low-mortality countries. There was no substantial heterogeneity (*I*^2^ range: 0–36%). The median vaccine effectiveness in low-mortality countries was similar for Rotarix (83%; interquartile range (IQR) 78–91), RotaTeq (85%; 81–92), mixed series (86%; 70–91), and non-product-specific (89%; 75–91) vaccination [15].

Our findings indicate that most of these trials were conducted in infants, which is in line with the need to understand the risk of intussusception in this population, especially when low-income countries are looking for or have introduced rotavirus vaccine in their immunisation schedules [4, 9]. While young children have been shown to have an increased risk of intussusception, a few rotavirus outbreaks among adults have been reported in the literature [6]. An example is rotavirus infection in immunocompromised adults, which can vary from symptomless to severe [6]. Our data also show several studies where populations older than infants have been included in the clinical trials.

We further show that 64% of rotavirus vaccine trials were registered with ClinicalTrials.gov, followed by CTRI (14%) and EU Clinical Trials Register (12%), WHO primary registers. Conversely, the continents in which these rotavirus vaccine trials have recruitment sites in Asia (41%) and North America (20%), with the maximum number of trials in the clinical trial registries coming from India (21%) and the USA (11%) with most being sponsored by the pharmaceutical industry. The findings align with the reports that post-licensure surveillance had been conducted in the countries in which rotavirus vaccines were introduced in national immunisation programmes [4, 16–18].

Our data also show that, while post-licensure surveillance was ongoing, new vaccines were also being tested to add to the already licenced vaccine, with 33% of the rotavirus vaccine trials in phase 3 clinical stages spread across from the year 2004 to 2020. Only 26% of the rotavirus vaccine trials indicated the availability of results, with most not yet recruiting. The unavailability of trial results in the clinical trials registry can impede researchers’ ability to assess all the available evidence on clinical trials, inform new research, and ultimately be used for policy development. Our analysis shows that there is a need to advocate for continual clinical trial registry data to be updated, thus promoting sharing the most updated data on the ongoing clinical trial research [12–14]. Furthermore, clinical trial registries can enable clinical trial data sharing to advance the use of evidence for new research ideas to fill existing gaps. At the same time, also track ongoing studies to be used as part of evidence generation. In the context of the rotavirus vaccine, the availability of clinical trial data could be beneficial for other low-income countries to understand how other countries, especially those with post-licensure trials, draw lessons learned from rotavirus vaccine introduction into the national immunisation programmes.

## Conclusion and future implications

Mapping rotavirus vaccine clinical trial activity provides useful information on planned, ongoing, or completed trials for researchers, funders, and healthcare decision-makers. Despite the high rotavirus disease burden in low- and middle-income countries including Africa, there is minimal clinical trial activity on the continent related to the condition. Clinical trial registries serve as a useful tool to share interim results of the trials especially those in the post-licensure stage to add to the understanding of the introduction of rotavirus vaccines into national immunisation programme for those countries that are yet to include rotavirus vaccines in their immunisation programmes given the risk of intussusception in children. Continued research on rotavirus vaccines is vital to comprehend the work required to implement new vaccines in national programmes. Clinical trial registration can be used to share findings from these studies; thus, it is crucial to keep the information in registries updated. Moreover, researchers can capitalise on the registries to share their results while waiting for the peer review process to get a publication.

## Data Availability

The data used to prepare this manuscript and the analysis is available on request.
